# Two Chikungunya Isolates from the Outbreak of La Reunion (Indian Ocean) Exhibit Different Patterns of Infection in the Mosquito, *Aedes albopictus*

**DOI:** 10.1371/journal.pone.0001168

**Published:** 2007-11-14

**Authors:** Marie Vazeille, Sara Moutailler, Daniel Coudrier, Claudine Rousseaux, Huot Khun, Michel Huerre, Julien Thiria, Jean-Sébastien Dehecq, Didier Fontenille, Isabelle Schuffenecker, Philippe Despres, Anna-Bella Failloux

**Affiliations:** 1 Département de Virologie (Chikungunya program), Institut Pasteur, Paris, France; 2 Génétique moléculaire des Bunyavirus, Institut Pasteur, Paris, France; 3 Cellule d'Intervention Biologique d'Urgence, Institut Pasteur, Paris, France; 4 Histotechnologie et Pathologie, Institut Pasteur, Paris, France; 5 Direction Régionale des Affaires Sanitaires et Sociales de La Réunion, Saint-Denis, France; 6 Institut de Recherche pour le Développement, UR 016 Caractérisation et Contrôle des Populations de Vecteurs, BP 64501, Montpellier, France; 7 Centre National de Référence des Arbovirus et Virus des Fièvres hémorragiques, Lyon, France; 8 Interactions Moléculaires Flavivirus-Hôtes, Institut Pasteur, Paris, France; U.S. Naval Medical Research Center Detachment/Centers for Disease Control, United States of America

## Abstract

**Background:**

A Chikungunya (CHIK) outbreak hit La Réunion Island in 2005–2006. The implicated vector was *Aedes albopictus*. Here, we present the first study on the susceptibility of *Ae. albopictus* populations to sympatric CHIKV isolates from La Réunion Island and compare it to other virus/vector combinations.

**Methodology and Findings:**

We orally infected 8 *Ae. albopictus* collections from La Réunion and 3 from Mayotte collected in March 2006 with two Chikungunya virus (CHIKV) from La Réunion: (i) strain 05.115 collected in June 2005 with an Alanine at the position 226 of the glycoprotein E1 and (ii) strain 06.21 collected in November 2005 with a substitution A226V. Two other CHIKV isolates and four additional mosquito strains/species were also tested. The viral titer of the infectious blood-meal was 10^7^ plaque forming units (pfu)/mL. Dissemination rates were assessed by immunofluorescent staining on head squashes of surviving females 14 days after infection. Rates were at least two times higher with CHIKV 06.21 compared to CHIKV 05.115. In addition, 10 individuals were analyzed every day by quantitative RT-PCR. Viral RNA was quantified on (i) whole females and (ii) midguts and salivary glands of infected females. When comparing profiles, CHIKV 06.21 produced nearly 2 log more viral RNA copies than CHIKV 05.115. Furthermore, females infected with CHIKV 05.115 could be divided in two categories: weakly susceptible or strongly susceptible, comparable to those infected by CHIKV 06.21. Histological analysis detected the presence of CHIKV in salivary glands two days after infection. In addition, *Ae. albopictus* from La Réunion was as efficient vector as *Ae. aegypti* and *Ae. albopictus* from Vietnam when infected with the CHIKV 06.21.

**Conclusions:**

Our findings support the hypothesis that the CHIK outbreak in La Réunion Island was due to a highly competent vector *Ae. albopictus* which allowed an efficient replication and dissemination of CHIKV 06.21.

## Introduction

First isolated in Tanzania in 1952 [1], Chikungunya virus (CHIKV) is a zoonotic arthropod-borne virus (*Alphavirus* genus, Togaviridae family) endemic to Africa, India and South-East Asia. In Africa, the virus is maintained within a sylvatic cycle with wild mosquitoes (*Aedes furcifer*, *Aedes luteocephalus*, *Aedes taylori*, *Aedes africanus*) feeding preferentially on primates (*Cercopithecus aethiops*, *Papio papio* and *Erythrocebus patas)* [2], [3]. In Asia, CHIKV is mainly transmitted within an urban cycle in an inter-human transmission achieved essentially by the human-biting *Aedes aegypti*, which breeds in man-made sites, and the less anthropophilic *Aedes albopictus*, which prefers suburban and rural areas where it colonizes both artificial and natural containers [4], [5]. Re-emergence of Chikungunya (CHIK) outbreaks is unpredictable and occurs frequently after 7–8 years of silence: Africa in 1999 in Kinshasa [6] and Asia in Java in 2001 [5]. At the end of 2004, CHIK has emerged in the Indian Ocean and was responsible of at least 266,000 cases on La Réunion Island.

Considered to be a secondary vector, *Ae. albopictus* (Skuse), the Asian “tiger mosquito”, is involved in the CHIK outbreak in the Indian Ocean in 2005–2006. This species native from South-East Asia [7] has spread as far West as Madagascar and most islands in the Indian Ocean and East through the Indomalayan and Oriental regions. The distribution of *Ae. albopictus* has expanded recently invading temperate zones such as the United States and Southern Europe, and is currently invading African countries [8]. *Ae. albopictus* is a competent laboratory vector for numerous arbovirus [9]. Vector competence which refers to the intrinsic permissiveness of a vector to transmit a pathogen is measured in laboratory by estimating oral susceptibility of mosquitoes using an artificial feeding protocol. Thus, *Ae. albopictus* has been demonstrated to be more susceptible to the African genotype of CHIKV than *Ae. aegypti* [10], [11], [12], [13]. In La Réunion Island, after intensive DDT treatments for malaria control in the 1950s, *Ae. aegypti* became rare [14], [15]. The decline in *Ae*. *aegypti* populations was associated with *Ae. albopictus* infestation of unoccupied breeding sites. In 1977, *Ae. albopictus* was responsible of a major dengue 2 outbreak in La Réunion Island affecting 30 to 35% of the population [16], [17]. In La Réunion, no animal reservoirs have yet been identified for CHIKV and only a human-vector-human cycle is described.

Phylogenetic analyses based on partial E1 sequences revealed the existence of three distinct phylogroups for CHIKV: one with the West African isolates, another including the Asian isolates and one regrouping the Eastern, Central and South African isolates [18]. Recent phylogenetic studies based on 126 E1 sequences from viral strains of the Indian Ocean 2005–2006 outbreak showed that these CHIKV strains belonged to the Eastern-Central-South African phylogroup [19]. Noteworthy, it has been observed that a single C to T non-synonymous substitution at the position 10670 was observed in some isolates. This nucleotide change was mapped in the E1 ectodomain. Indeed, CHIKV E1-226 genotype swapped during the winter season 2005 in the Indian Ocean: whereas E1-Ala226 was typically observed in CHIKV isolates during the first period of the outbreak (before September 2005), E1-Val226 was present in E1 sequences in more than 90% of viral strains isolated during the second period (December 2005 to March 2006). We took advantage that CHIKV 05.115 and 06.21 differ by the single E1 substitution to evaluate whether the A226V change had an impact on viral replication in vectors.

In the present study, we showed that (i) examined populations of *Ae. albopictus* from La Réunion and Mayotte exhibited differential susceptibilities to La Réunion CHIKV isolates, (ii) CHIKV 05.115 replication was restricted when compared to CHIKV 06.21, (iii) although both CHIKV 05.115 and CHIKV 06.21 invaded salivary glands in a similar pattern, the crossing of midgut was the critical step in the susceptibility of *Ae. albopictus* to CHIKV isolates, (iv) females infected with CHIKV 05.115 could be divided in two categories: weakly susceptible or strongly susceptible, comparable to those infected by CHIKV 06.21 and (v) *Ae. albopictus* from La Réunion Island and Asian CHIKV vectors showed similar ability to support CHIKV 06.21 replication.

## Materials and Methods

### Mosquitoes

Eight mosquito samples were collected in La Réunion Island and three in Mayotte in March 2006. All collections were mainly composed of *Ae. albopictus*. The collections STPIE2 and STPIE3 contained in addition *Culex quinquefasciatus* and the collection MAYOT1, *Ae. aegypti* which has not been tested as no progeny could be obtained (see Table 1). The mosquitoes collected as larvae and/or pupae in breeding sites were brought back to laboratory and reared until adult stage (F0 generation) at 28±1°C with 80% relative humidity and a 16 h:8 h photoperiod. Adults were given 10% sucrose solution and females were allowed to feed every two days on a mouse to obtain eggs. To obtain enough females of the same physiological age for oral infections, batches of eggs were hatched and larvae reared to the adult stage (F1 generation) in pans with tap water and yeast tablets. One week-old F1 females were tested for their susceptibility to CHIKV infection. The Paea strain of *Ae. aegypti* provided by Institut Louis Malardé (Tahiti, French Polynesia) and reared in Paris since 1994, was used as a control of mosquito susceptibility.

**Table 1:** Characteristics of mosquito collections carried out in March 2006.

Collection	Collection		Breeding site	Species collected					
	Date	Site		*Aedes albopictus*		*Culex quinquefasciatus*		*Aedes aegypti*	
				Females	Males	Females	Males	Females	Males
**La Réunion**									
STAND	10/03/2006	Saint-André	Tree hole	35	25	-	-	-	-
STBEN	10/03/2006	Saint-Benoit	Bamboo hole	44	32	-	-	-	-
STDEN	10/03/2006	Saint-Denis	Vase	70	68	-	-	-	-
STPAU1	10/03/2006	Saint-Paul	Vase	11	11	-	-	-	-
STPAU2	09/03/2006	Saint-Paul	Vase	18	18	-	-	-	-
STPIE1	09/03/2006	Saint-Pierre	Rock hole	1	6	-	-	-	-
STPIE2	09/03/2006	Saint-Pierre	Tyres	40	39	20	14	-	-
STPIE3	09/03/2006	Saint-Pierre	Bucket	25	44	13	8	-	-
**Mayotte**									
MAYOT1	10/03/2006	Kavani	Various artificial containers (cans, bottles..)	60	60	-	-	10	8
MAYOT2	10/03/2006	Kavani	Various artificial containers (cans, bottles..)	68	63	-	-	-	-
MAYOT3	10/03/2006	Kavani	Tyre	26	22	-	-	-	-

To compare the susceptibility status of *Ae. albopictus* from La Réunion, we also used other mosquito strains: (i) a colony STDEN1-F2 which derived from the field-collected population STDEN, (ii) *Ae. albopictus* MAYOT1-F1, (iii) the F1 generation *Ae. albopictus* and *Ae. aegypti* collected in Yaoundé, Cameroon in May 2006 (YAOUNDE-F1), (iv) a colony of *Ae. aegypti* (HCM) from Ho Chi Minh City, Vietnam maintained in laboratory for several years, and (v) a colony of *Ae. albopictus* (HANOI-F3) from Hanoi, Vietnam maintained for 3 generations in laboratory.

### Viruses

The different CHIKV isolates provided by the French National Reference Center for Arbovirus in Lyon have been entirely sequenced [19]. All the four strains were isolated on *Ae. albopictus* cells C6/36 [20] from human serum: (i) strain 05.115 in June 2005 from a 24-year old female from La Réunion presenting classical CHIK symptoms, (ii) strain 06.21 in November 2005 from a new-born male from La Réunion presenting meningo-encephalitis symptoms, (iii) strain 06.111 collected in February 2006 from a patient from Mayotte presenting classical CHIK symptoms; and (iv) strain 06.117 collected during the 1999–2000 outbreak in the Democratic Republic of Congo identified as a member of Eastern/Central/Southern African group [6]. CHIKV 05.115 isolated at the beginning of the outbreak had E1-226A and CHIKV 06.21 isolated later in the outbreak had E1-226V [19]. CHIKV 06.111 contained the change A->V in E1-226 [19] and CHIKV 06.117 has an Alanine at the position 226. Compared to the three other strains, CHIKV 06.117 had a change at the position 284 in the E1 glycoprotein from an Asp to a Glu. *Ae. albopictus* cells C6/36 were infected at a MOI of 5 and maintained at 28°C on L-15 medium supplemented with 10% fetal bovine serum (FBS), 1000 units/mL penicillin, 1 mg/mL streptomycin, and Tryptose phosphate broth 1×. Cell infection was checked by indirect immunofluorescence assays (IFA) using mouse ascitic fluid directed against CHIKV. Cells were fixed with methanol/acetone (7∶3) on glass spots at −20°C for 20 min. The fixed cells were incubated with specific ascitic fluids at a dilution of 1∶200 in PBS 1× at 37°C for 20 min. After washing with PBS 1×, cells were incubated at 37°C for 20 min with FITC-conjugated goat anti-mouse IgG antibody (Sigma) at a 1∶100 dilution in PBS 1×. Slides were examined using a fluorescence microscope. When 80% of cells were infected, the supernatant fluid was collected and viral titer estimated by serial 10-fold dilutions on Vero cells. Briefly, cells were incubated for 3 days under an overlay consisting of DMEM (Dulbecco's modified Eagle's medium), 2% FBS, antibiotics and 1% Indubiose (IBF Biotechnics) at 37°C. The lytic plaques were counted after staining with a solution of crystal violet (0.2% in 10% formaldehyde and 20% ethanol). Viral stocks which have been constituted after two passages on C6/36 cells were divided into aliquots and stored at −80°C until used. The genotypic characteristics of CHIKV inoculums have been verified by sequencing.

### Oral infection of mosquitoes

To calculate the viral titer to be used in the blood mixture of infection assays, different batches of *Ae. albopictus* STDEN1-F2 collected in La Réunion in 2006 were infected with different viral titers: 10^5^, 10^6^, 10^7^, 10^8^, and 10^9^ pfu/mL and dissemination rates were estimated. In addition, 30 females which had fed on a non-infected blood-meal were killed immediately after complete engorgement. Each individual mosquito was ground in Drabkin's solution according to a protocol described in Briegel et al. [21] to determine the quantity of blood ingested per female.

Infection assays were performed with 7 day-old females which were allowed to feed for 15 min through a chicken skin membrane covering the base of a glass feeder containing the blood-virus mixture maintained at 37°C. The infectious meal was composed of a virus suspension diluted (1∶3) in washed rabbit erythrocytes isolated from arterial blood collected 24 h before the infectious meal [22]. A phagostimulant ATP was added at a final concentration of 5× 10^−3^ M. Fully engorged females were transferred to small cardboard containers and maintained with 10% sucrose at 28±1°C for 14 days. To evaluate dissemination rate and thus vector competence, surviving females were frozen at −80°C and tested for the presence of CHIKV antigens in head squashes by IFA.

To estimate the number of RNA copies and identify the preferential replication site of the virus in mosquitoes, batches of 15 *Ae. albopictus* STDEN1-F2 were sacrified every day post-infection (pi): 10 individuals were used for quantitative RT-PCR and 5 for histology. For quantitative RT-PCR, 5 mosquitoes were dissected to isolate the midgut and the salivary glands, and 5 were used to measure the number of RNA copies in the whole female.

### Quantitative RT-PCR analysis

Total RNA was extracted using the Nucleospin® RNA II kit (Macherey-Nagel) following the manufacturer's instructions. Briefly, individual mosquito was ground in 350 µl of lysis buffer and 3.5 µl of β-mercaptoethanol. The lysate was then filtered through filter units and centrifuge for 1 min at 11,000 g. The filtrate collected in a tube was mixed with ethanol 70%. The solution was passed through a column which binds RNA after centrifugation for 30 s at 8,000 g. After desalting the silica membrane (centrifugation at 11,000 g for 1 min), a DNAse reaction mixture was applied on the silica membrane of the column for 15 min at room temperature. After different cycles of washing, the RNA solution was eluted by centrifugation at 11,000 g for 1 min in RNAse-free H_2_0.

To build the standard curve, a CHIKV RNA synthetic transcript was generated. A PCR product encompassing the targeted region was prepared using the CHIKV and cloned into pCR II TOPO vector (Invitrogen). The amplified product using vector-specific primers was purified using the PCR Purification Kit (Qiagen). RNA transcripts were produced *in vitro* using the RiboMAX™ Large Scale RNA Production Systems (Promega) appropriate for either SP6 or T7 RNA polymerase. The transcript size was 1,356 bp for both CHIKV 05.115 and CHIKV 06.21. Residual DNA has been eliminated by several DNAse treatments (Turbo DNA-free (Ambion)). After quantification by spectrophotometer, RNA transcript solution was stored at −80°C.

The one-step RT-PCR was performed in a volume of 25 µl containing 3 µl RNA template, 12.5 µl 2× Brilliant SYBR Green I QPCR Master Mix (Stratagene), 1 µl sense (2.5 µM), 1 µl anti-sense (2.5 µM), 0.25 µl Fluorescein (1 µM), and 0.0625 µl Stratascript RT/RNAse block enzyme. Primers were selected in the E2 structural protein regions of sequences retrieved from the GenBank database by the Laboratory for Urgent Response to Biological Threats at the Institut Pasteur: sense Chik/E2/9018/+ (CACCGCCGCAACTACCG) and anti-sense Chik/E2/9235/- (GATTGGTGACCGCGGCA). The amplification program in a i-Cycler™ (Biorad) included: a reverse transcription at 50°C for 30 min, an inactivation step of RT/RNAse enzyme at 95°C 10 min followed by 40 cycles of 95°C 30 s, 56°C 1 min, 72°C 30 s, a step at 95°C 1 min, and 81 cycles of 55°C (+0.5°C/cycle) 30 s. The size of the amplification product was 217 bp. After amplification, a melting curve was acquired to check the specificity of PCR products. PCR was performed in triplicate for each mosquito and five mosquitoes were tested simultaneously every day post-infection (pi). Signals were normalized to the standard curve using serial dilutions of RNA synthetic transcripts. Normalized data were used to measure the number of RNA copies in infected mosquitoes according to the ΔC_t_ analysis.

### Histological examinations

Every day after infection, 5 females were killed and fixed in Carnoy solution (3 vol. chloroform, 1 vol. absolute ethanol, 1 vol. acetic acid). Samples were then dehydrated as follows: 8 h in absolute ethanol, 17 h in solution 1 (55% n-butanol/40,5% absolute ethanol in H_2_O), 8 h in solution 2 (75% n-butanol/22.5% absolute ethanol in H_2_O) and finally 2–3 days in n-butanol. Mosquitoes were embedded in Paraffin. Sections (5 µm) were stained with hematoxylin and eosin, periodic acid Schiff, and Gordon sweet stains according to Bancroft et al. [23]. Immunohistochemical analysis was performed by using a polyclonal mouse ascitic fluid at a dilution 1∶750. Briefly, tissue sections were immersed in 200 mL of citrate and incubated three times for 5 min in a microwave at 650 W before staining. The streptavidin peroxydase method with AEC (amino ethyl carbozole) as a chromogen was used to detect the secondary antibody (Envision system labeled Polymer-HRP antimouse, Dako). Slides were counterstained with Meyer's hematoxylin. Positive slide controls were provided from CHIKV-C636 infected cells included in an avian muscle and fixed in formalin then embedded in paraffin blocks. Negative controls included both uninfected C6/36 cells treated by the same protocol and slides from uninfected mosquitoes which have taken a blood-meal without CHIKV. Slides were examined by light microscopy.

### Statistical analysis

Variations in the percentages of engorged females and females with disseminated infection at respectively, day 0 and day 14 pi were compared using the RxC Fisher's exact test [24].

## Results

**Figure 1:**
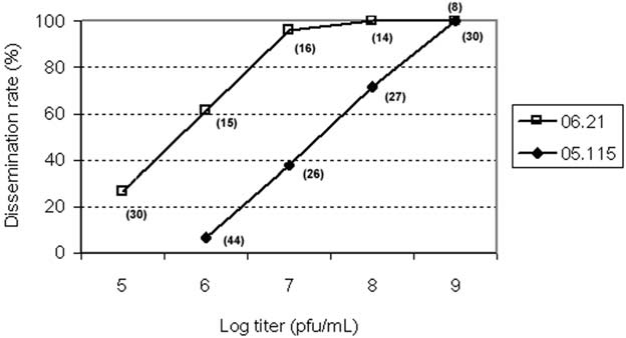
Dissemination rates of *Aedes albopictus* infected with CHIKV 05.115 and CHIKV 06.21 at different viral titers. In brackets, the number of females tested is given.

To evaluate the susceptibility of *Aedes* mosquitoes to CHIKV infection, batches of STDEN-F2 females were infected with CHIKV strains 05.115 and 06.21 using an artificial infectious blood-meal. At day 14 pi, IFA on head squashes of infected mosquitoes showed a linear progression between dissemination rates and CHIKV titers (Figure 1). STDEN-F2 females showed increased susceptibility to CHIKV strain 06.21 as compared with mosquitoes infected with 05.115. Blood meal with 10^7^ pfu/mL of CHIKV 06.21 was sufficient to infect 96% of females while 10^7^ pfu/mL of CHIKV 05.115 resulted in 37.5% infection. The titer 10^7^ pfu/mL was discriminant enough to differentiate dissemination rates between CHIKV isolates 05.115 and 06.21, and was chosen to infect field-collected *Ae. albopictus*. Ten individuals were collected immediately after blood-meal and the viral titer for each female was estimated by plaque assay on Vero cells. The titer per female was 10^4.0^ (±10^0.12^) with CHIKV 05.115 and 10^5.03^ (±10^0.31^) with CHIKV 06.21. Using the Drabkin's method, the quantity of blood ingested by an uninfected female was 4.15 µl (±2.48). Thus the number of viral particles ingested per female should be 10^4.6^ pfu. However, a difference of 0.5 log for each virus was found when compared to the theoretical value: −0.5 for CHIKV 05.115 and +0.5 for CHIKV 06.21. This discrepancy has already been reported in the literature [12] using this same technique of titration.

### How variable is the vector competence of *Ae. albopictus* from La Réunion and Mayotte islands? (Table 2)

**Table 2:** Dissemination rates of *Aedes albopictus* from La Réunion and Mayotte evaluated at day 14 post-infection with CHIKV (isolates 05.115 and 06.21) (control: *Ae*. *aegypti* Paea strain from Tahiti).

Site	Collection	05.115		06.21		*P*
		Assay	Control	Assay	Control	
**LA REUNION**						
Saint-André	STAND	26.9 (26)	43.5 (69)	87.5 (32)	94.9 (39)	**10^−4^**
Saint-Benoit	STBEN	20.0 (35)	43.5 (69)	88.7 (53)	94.9 (39)	**10^−4^**
Saint-Denis	STDEN	37.3 (59)	30.2 (43)	95.8 (24)	90.5 (21)	**10^−4^**
Saint-Paul	STPAU1	33.3 (15)	43.5 (69)	100 (27)	94.9 (39)	**10^−4^**
	STPAU2	29.3 (58)	44.7 (38)	100 (60)	98 (51)	**10^−4^**
	STPAU	30.14 (73)^*^		100 (87)^*^		**10^−4^**
Saint-Pierre	STPIE1	33.3 (6)	44.7 (38)	100 (8)	98 (51)	**0.014**
	STPIE2	20.4 (54)	43.5 (69)	94.0 (50)	94.9 (39)	**10^−4^**
	STPIE3	10.5 (38)	44.7 (38)	80 (5)	98 (51)	**0.003**
	STPIE	17.3 (98)^*^		93.6 (63)^*^		**10^−4^**
**MAYOTTE**						
	MAYOT1	18.1 (83)	51.2 (121)	90.5 (74)	95.8 (119)	**10^−4^**
	MAYOT2	26.5 (83)	51.2 (121)	88.0 (50)	95.8 (119)	**10^−4^**
	MAYOT3	30.8 (65)	51.2 (121)	97.4 (38)	95.8 (119)	**10^−4^**
	MAYOT	24.7 (231)^*^		91.4 (162)^*^		**10^−4^**

In brackets, is given the number of females tested; *P*: Probability of homogeneity from Fisher's exact test. Significant values (P<0.05) are in bold. ^*^ data from collections of the same site have been pooled.

Mosquitoes showed dissemination rates ranging from 10.5% (STPIE3) to 37.3% (STDEN) when infected with CHIKV 05.115 and from 80% (STPIE3) to 100% (STPAU1, STPAU2, STPIE1) when infected with CHIKV 06.21. The control *Ae. aegypti* Paea strain showed dissemination rates which ranged from 30.2% to 51.2% for CHIKV 05.115 and from 90.5% to 98% for CHIKV 06.21. When comparing collections from the same site (Saint-Paul, Saint-Pierre and Kavani, see Table 1), no significant difference was found between dissemination rates. Thereby, data from the same collection site were pooled (Table 2). When comparing for each collection the percentage of females infected with CHIKV 05.115 with the percentage of females infected with CHIKV 06.21, significant differences were obtained (Fisher's exact test: P>0.05).

### Is *Ae. albopictus* a good amplifier of CHIKV?

The number of RNA copies in mosquitoes was estimated every day pi. The standard curve calculated from serial dilutions of RNA synthetic transcripts in triplicate, was linear over 9-log range (from 10^1^ to 10^9^ copies).

#### Quantification of CHIKV in whole females (Figure 2)

**Figure 2:**
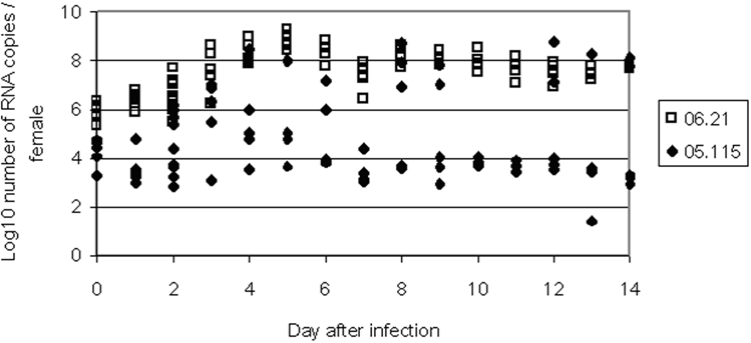
Viral replication in whole females of *Aedes albopictus* after oral infection with CHIKV 05.115 and CHIKV 06.21.

For CHIKV 05.115, after a small peak at 10^5^ RNA copies/female at days 4 and 5, the number of RNA copies stabilized between 10^3^ and 10^4^ until day 14 for the majority of females. However, a high variation was observed between the 5 analyzed females, some of them replicating CHIKV 05.115 as efficiently as CHIKV 06.21. For CHIKV 06.21, the number of RNA copies increased regularly between day 1 and day 5 from 10^6^ to 10^8–9^ RNA copies/female. After a small drop from day 5 to day 7, the number of RNA copies persisted roughly at 10^7^–10^8^ until day 14 pi. Variation between values of the 5 females tested each day was very low. When comparing the two profiles, CHIKV 05.115 was nearly 2 log lower than CHIKV 06.21.

#### Quantification of CHIKV in midguts (Figure 3A)

**Figure 3:**
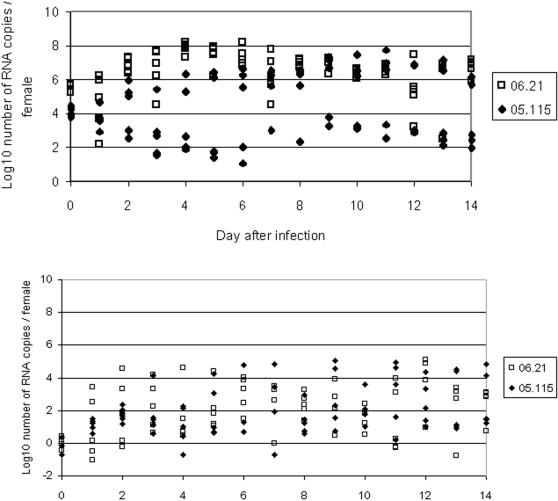
Quantification of CHIKV 05.115 and CHIKV 06.21 of *Aedes albopictus.* *(A)* midguts. *(B)* salivary glands

For CHIKV 05.115, when considering the number of RNA copies evaluated in midguts, two categories of females could be distinguished: (1) females which replicated at a low level (lower than 10^4^ RNA copies/female) and (2) females which replicated at a level similar to females infected with CHIKV 06.21 (higher than 10^6^ RNA copies/female). For CHIKV 06.21, the number of RNA copies increased from 10^5^–10^6^ to reach a maximum (10^7^–10^8^) at day 4 pi and then, decreased very slowly with a minimum (10^6^–10^7^) at day 12 pi.

#### Quantification of CHIKV in salivary glands (Figure 3B)

At each time point, the number of RNA copies evaluated in salivary glands was highly variable and could not allow to distinguish the two viral strains. Values were dispersed from 0 to 10^5^ RNA copies per salivary glands.

#### Histological examination (Figure 4)

**Figure 4:**
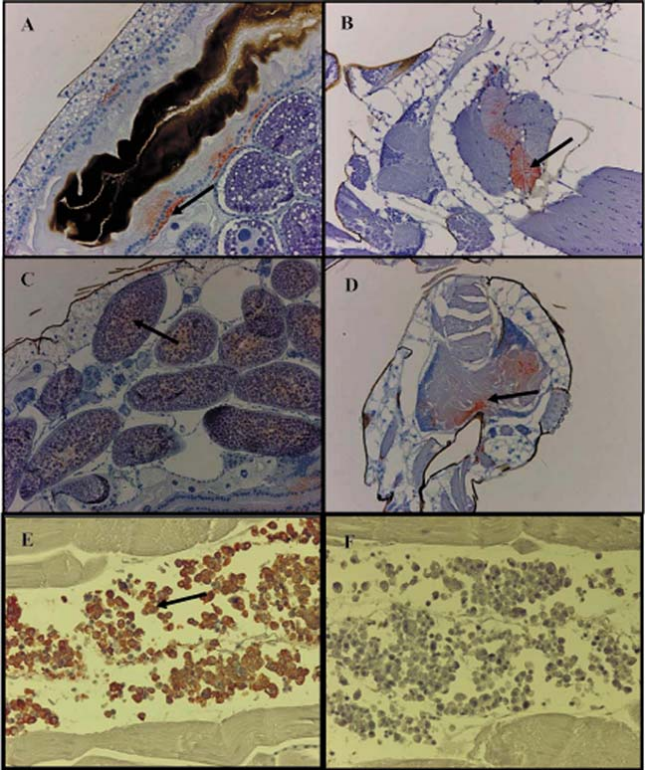
Immunocytochemical preparations of *Aedes albopictus* tissues infected with CHIKV 06.21. (A) midgut day 1 pi. (B) salivary glands day 2 pi. (C) ovaries day 6 pi. (D) nervous central system day 9 pi. (E) CHIKV-infected C6/36 cells as positive control. (F) non infected C6/36 cells as negative control. Magnification: ×100.

Slides showed that one day after ingestion of the infected blood-meal, the virus colonized the epithelial cells of the midgut (Figure 4A). At day 2 pi, the virus was also visible in the salivary glands (Figure 4B) and at day 6, eggs became infected (Figure 4C). During the 14 days of observation, the midgut remained infected. From day 9 until day 14 pi, the midgut, the salivary glands, the ovaries and the central nervous system (Figure 4D) were infected. Cells from the different tissues did not seem to be damaged by viral infection.

### How efficient is the couple *Ae. albopictus* and CHIKV in La Réunion compared with other vector/CHIKV combinations? (Table 3)

**Table 3:** Dissemination rates of *Aedes albopictus* and *Aedes aegypti* estimated 14 days post-infection with different CHIKV isolates (La Réunion, Mayotte, and Democratic Republic of Congo).

Viral strains	*Aedes albopictus*				*Aedes aegypti*	
	STDEN-F2	MAYOT1-F1	YAOUNDE-F1	HANOI-F3	YAOUNDE-F1	HCM
La Réunion						
05.115	37.5 (16)	18.10 (83)	12.20 (90)	29.60 (54)	37.10 (97)	66.5 (206)
06.21	100 (25)	90.5 (74)	68.30 (60)	94.70 (57)	64.50 (107)	96.6 (234)
Mayotte						
06.111	96.80 (94)	98 (49)	77.30 (44)	84.10 (126)	82.90 (82)	92.0 (137)
Democratic Republic of Congo						
06.117	80.60 (31)	73.20 (56)	56.40 (39)	47.10 (119)	84.80 (66)	78.30 (138)
*P*	**<10^−4^**	**<10^−4^**	**<10^−4^**	**<10^−4^**	**<10^−4^**	**<10^−4^**

In brackets, is given the number of females tested.

*P*: Probability of homogeneity from Fisher's exact test. Significant values (P<0.05) are in bold.

When comparing dissemination rates of the different mosquito collections obtained with the four CHIKV isolates (05.115, 06.21, 06.111 and 06.117), they were significantly different (P<10^−4^). *Ae. albopictus* STDEN1-F2 and HANOI-F3 gave the highest dissemination rates when infected with CHIKV 06.21 (respectively, 100% and 94.70%). However, MAYOT1-F1 and YAOUNDE-F1 were more susceptible to CHIKV 06.111 (respectively, 98% and 77.30%). *Ae. albopictus* STDEN-F2, MAYOT1-F1, HANOI-F3 and HCM displayed similar dissemination rates when infected with CHIKV 06.21 (Fisher's exact test: P = 0.131). The strain STDEN-F2 showed similar infections rates towards CHIKV 06.21 and CHIKV 06.111.

## Discussion

In 2005–2006, CHIKV has caused one of the largest CHIK outbreaks in the world affecting at least one third of the population in La Réunion Island. Whereas CHIKV often circulated in Africa and Asia, it has never been reported in the Indian Ocean and in La Réunion Island where *Ae. albopictus* has been incriminated. The vector competence for four CHIKV isolates has been assessed in different mosquito vectors including *Ae. albopictus* from La Réunion. We found that (i) the CHIKV 06.21 strain gives higher dissemination rates and better replicates in *Ae. albopictus* from La Réunion, (ii) the midgut plays a key role in viral replication, and (iii) *Ae. albopictus* from La Réunion is as efficient vector as *Ae. aegypti* and *Ae. albopictus* from Vietnam when infected with the CHIKV 06.21.

### Is CHIKV 06.21 more efficiently transmitted by *Ae. albopictus*?

The main difference in amino-acids between the two viral strains isolated from La Réunion Island is the change at the position 226 of the glycoprotein E1 from an Alanine (CHIKV 05.115 collected in June 2005 at the beginning of the outbreak) to a Valine (CHIKV 06.21 collected in November 2005 later in the outbreak) [19]. As a change at residue 226 in another alphavirus, the Semliki Forest virus, has been shown to be involved in the membrane fusion [25], [26], it has been assumed that the A226V change could favour infection of mosquito cells. When infecting *Ae*. *albopictus* from La Réunion, the dissemination rates at 14 days pi were different between the two CHIKV: most of mosquitoes infected with CHIKV 06.21 allowed an efficient viral dissemination whereas less than a half when infected with CHIKV 05.115. Numerous experimental transmission studies with *Ae. aegypti* and *Ae. albopictus* demonstrated their high capacity to transmit CHIKV [13]. The titer of 10^7^ pfu/mL we used to infect our mosquito collections has been chosen to better distinguish CHIKV 05.115 and CHIKV 06.21. As all pathogens transmitted by mosquitoes, CHIKV is acquired with a blood-meal. Two physical barriers can affect its transmission: the midgut and the salivary glands [27]. Based on our results, CHIKV 05.115 and CHIKV 06.21 could be distinguished by the number of RNA copies estimated by quantitative RT-PCR. CHIKV 06.21 replicated at a high level and homogenously whereas CHIKV 05.115 showed two distinct profiles: most of females ensured a low replication level and only a few replicated CHIKV 05.115 as efficiently as CHIKV 06.21. Binding to putative virus-specific receptors present in the brush border membrane of the midgut epithelial cells appears to mediate the attachment and the entry of the virus into midgut cells [28]. These proteins are present in both CHIKV-susceptible and -refractory mosquitoes. However, the binding efficacy is greater in susceptible than refractory mosquitoes. In our study, the midgut appeared infected during the whole incubation time. The midgut could be involved as a mesenteronal escape barrier acting in a way to limit virus dissemination into the hemocoele and thus preventing dissemination altogether [29]. So, once CHIKV 05.115 succeeds in crossing the midgut barrier, it replicates as efficiently as CHIKV 06.21. However, the proportion of such phenotype is low explaining the low dissemination rates obtained. The relative similarity of infection between the two CHIKV in salivary glands is consistent with the idea that once virus escapes from the midgut, its spread to other tissues is very fast and homogeneous. In our case, the virus was present in salivary glands 2 days after an infective blood-meal. So CHIKV appeared to have a short incubation period enabling *Ae. albopictus* to transmit the virus as early as two days after an infective blood-meal. This has already been observed with other arboviruses such as Rift valley fever virus [30] and could probably explain the high transmission of CHIKV in some foci in La Réunion Island (Thiria, personal communication).

### General considerations on CHIK transmission

CHIKV has been introduced into a region where the human herd immunity was minimal and where *Ae. albopictus*, a secondary CHIKV vector proliferated. Phylogenetic analyses based on partial glycoprotein E1 sequences indicate that the Indian Ocean outbreak was caused by the same strain in La Réunion Island, Seychelles, Mayotte, Madagascar, and Mauritius [19]. These isolates represent a homogeneous clade within a group of viral isolates from East, Central and South Africa. The CHIKV isolated from the last urban outbreak in Kinshasa (Democratic Republic of Congo) in 1999–2000 belonged to the Central African lineage [6]. The analyzed CHIKV 06.117 showed highest dissemination rates when infecting *Ae. aegypti* rather than *Ae. albopictus*. In Kinshasa, *Ae. aegypti*, present in high densities [31] has been incriminated in CHIKV transmission. CHIKV 05.115, isolated in June 2005 in La Réunion at the beginning of the outbreak, was close to the African CHIKV S27 [32] isolated during the 1952 Tanzania outbreak leading Schuffenecker et al. [19]to assume that CHIKV 05.115 represents the ancestral genotype of La Réunion outbreak. It differed from the CHIKV 06.117 isolated in Democratic Republic of Congo principally by a change at the position 284 in the E1 glycoprotein from an Asp (CHIKV 06.117) to a Glu (CHIKV 05.115 and 06.21). Infection of *Ae. albopictus* with CHIKV 05.115 triggers an heterogeneous response in mosquitoes. While most mosquitoes did not allow active viral replication, few mosquitoes enabled replication and dissemination as efficiently as when infected with CHIKV 06.21. Thus dissemination rates in *Ae. albopictus* were lower with CHIKV 05.115. In La Réunion, *Ae. albopictus* colonizes domestic environments which enhances its contact with human beings leading the mosquito to feed almost exclusively on humans [33]. The species was involved in the dengue 2 outbreak of 1977 [17] and in the dengue 1 outbreak of April 2004 in La Réunion Island. Few months later, the CHIK outbreak began in La Réunion Island responsible, after a period of low transmission during the southern winter, of more than 266,000 cases and 255 deaths (data from the “Cire Réunion-Mayotte”, November 15^th^ 2005). After December 2005, most patients harboured essentially CHIKV isolates with an amino-acid change at the position E1-226V [19]. CHIKV 06.21 was very efficiently transmitted by *Ae. albopictus* from La Réunion. Has this mutation been selected as more adapted to an alternative, abundant mosquito, *Ae. albopictus*? Envelop glycoprotein mutations that facilitate transmission by mosquito vectors have also been incriminated in the emergence process of other arboviral diseases [34]. As the islands in the Indian Ocean attract each year thousands of tourists, it was not surprising that CHIKV strains were able to invade the entire region including Mayotte where CHIKV 06.111 has been isolated in February 2006. To confirm the role of the E1-A226V substitution in the CHIK emergence process in the Indian Ocean, further studies are necessary. Reverse genetics studies placing the E1-A226V mutation into cDNA clones are needed to test this hypothesis. Besides, given our histological data, another field of research is open. The amount of viral particles present in the ovaries could indicate the possibility of a vertical transmission of CHIKV in *Ae. albopictus* from La Réunion. If proven, this would have a major impact on the transmission of the virus in this area.
